# Insight into structure dynamics of soil microbiota mediated by the richness of replanted *Pseudostellaria heterophylla*

**DOI:** 10.1038/srep26175

**Published:** 2016-05-18

**Authors:** Yong-Po Zhao, Sheng Lin, Leixia Chu, JiangTao Gao, Saadia Azeem, Wenxiong Lin

**Affiliations:** 1 College of Life Sciences, Fujian Agricultural and Forestry University, Fuzhou 35002, China; 2 Key Laboratory of Crop Ecology and Molecular Physiology, Fujian Agriculture and Forestry University, Fuzhou 35002, China; 3 Fujian Provincial Key Laboratory of Agroecological Processing and Safety Monitoring, Fujian Agriculture and Forestry University, Fuzhou 35002, China

## Abstract

Consecutive monoculture of crops causes serious diseases and significant decline in yield and quality, and microbes in the rhizosphere are closely linked with plant health. Here we systematically studied the structure dynamics of soil microbiota in the monocropping system of *Pseudostellaria heterophlla.* The results illustrated that the successive cropping of *P. heterophylla* shifts the diversity and structure of microbial community in rhizosphere soil of *P. heterophylla*, showing that the diversity of microbial community in rhizosphere soil of *P. heterophylla* was decreased with the increase of planting years while the structure of microbial community became more deteriorative. Moreover, the population size of typical pathogens increased and the beneficial bacterial population decreased with the increasing years of monoculture, which resulted in the microecological imbalance in *P. heterophylla* rhizosphere, thereby caused serious replanting diseases in monocropping system. Our results suggested that structure dynamics of rhizosphere microbial communities were mediated by the richness of replanted *P. heterophylla,* and thus the replant disease result from the imbalanced microbial structure with a higher ratio of pathogens/beneficial bacteria in rhizosphere soil under monocropping regimes. This finding provides a clue to open a new avenue for modulating the root microbiome to enhance the crop production and sustainability.

*P. heterophylla* belonging to the family of *Caryophyllaceae* has been used as an important traditional Chinese medicine and therapeutic for over 3000 years. It is mainly produced and cultivated in a geo-authentic production zone located in Zherong county, Ningde city, Fujian province of southeast China. The herb is widely used for treating diseases such as lung and spleen tonic^1^. Owing to its medicinal and economic value, the practice of monocropping shows an expanding pattern in popularity, primarily due to government incentives, technological advances and market needs. However, *P. heterophylla* has severe consecutive monoculture problems, such as decline in productivity and quality. This phenomenon is also known as soil sickness or replant disease^2^,^3^,^4^. The attempt to eradicate and solve this problem, less desirable areas outside Zherong had been used for planting *P. heterophylla*. This inevitably caused poor quality. Therefore, it has become a top priority to elucidate the underlying mechanisms of consecutive monoculture problems, especially in the case of medicinal plant production.

Previous studies pointed out that there are possible three reasons for the consecutive monoculture problems: decline in soil fertility, the autotoxicity of root exudates and the shifts in the microbial community^5^. Among them, the autotoxicity issue has attracted considerable attention in studies of some crops in the monocropping system^6^,^7^,^8^. However, many studies have demonstrated that allelochemical substances produced by root are able to shape the rhizosphere microbiome by deterring or attracting certain microbial species^9^,^10^,^11^,^12^,^13^,^14^,^15^. Recently, many researchers have reported that autotoxic substances in root exudates are not toxic to plant directly, but cause micro-ecological imbalance and hence influence the growth of plant indirectly^16^,^17^,^18^,^19^.

The rhizosphere containing abundant microorganisms is the narrow zone of soil, and it can be influenced by root secretions^15^. The role of microbial communities is very crucial and important in plant or animal health. For instance, in the human gut, the effects of intestinal microbial communities on health are becoming increasingly apparent^20^. Similarly, the metagenome of microbial communities in the rhizosphere of plants is much larger than that of the plant, and it is also referred to as the plant’s second genome^13^.

The roles of root microbial communities are also apparently increasing as research in this area is progressing. It is possible to determine plant health on the basis of plant microbial communities. In recent years, the concept “root microbiome” (or rhizosphere microbiome) has been intensively introduced to describe the complexity of root microbial communities, which consist of the entire complex of rhizosphere associated microbes, genetic elements and their interactions^13^.

Our previous study found that pathogenic *F. oxysporum* f.sp. *heterophylla* in rhizosphere soil of *P. heterophylla* reproduces increasingly in a consecutive monoculture system because of the selection and mediation of root exudates^21^. However, *F. oxysporum* f.sp. *heterophylla* is just one kind of pathogen from microbial communities in the rhizosphere of *P. heterophylla.* The root of plants can shape the microbial community structure in their rhizosphere. So, we infer that in monoculture system, the shift of microbial communities is closely related to the replant disease. However, the effect on the status of the whole soil microbial communities in the rhizosphere of replanted *P. heterophylla* remains unclear. Here we study the shifts of the microbial community in the rhizosphere of replanted *P. heterophylla* to clarify the correlation between root-associated microbial community and successive cropping of *P. heterophylla* by using 16S rRNA amplicon metagenome sequencing and Denaturing Gradient Gel Electrophoresis (DGGE). Our study will further provide new insights into the mechanism of replant disease of *P. heterophylla* in monocropping system.

## Results

### The 16S rRNA amplicon metagenomic analysis of soil bacteria

**Figure 1:**
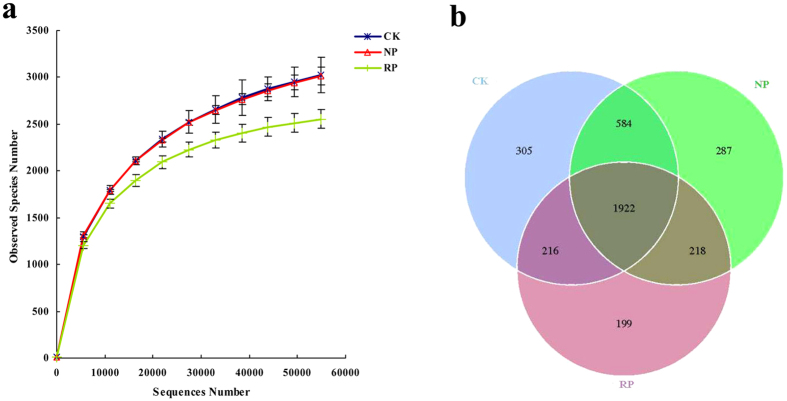
The Rarefaction Curve (**a**) and Venn Graph (**b**) based on 97% similarity. CK, RP, NP refer to control soil without planting any crop, newly planted soil, replanted soil, respectively. (**a**) Data are representative of 3 independent experiments ± s.d. The figure is representative of 3 independent experiments.

Rarefaction analysis (Fig. 1a) showed that the number of OTUs for 16S rDNA tended to plateau after 50,000 sequences at 97% similarity. This suggested that the sequencing result could relatively reflect the microbial diversity and richness of these soil samples accurately. General analyses of the sequencing data indicated that a total 336,548 reads for 16S rDNA could be paired successfully. After removing short and low-quality reads, singletons, replicates and chimeras, 323,715 sequences ranging from 67,447 to 129,990 per sample were retained for 16S rDNA. Among the total 16S rDNA sequences, 99% sequences were classified as bacteria, which included 39 phyla, whereas the remaining 1% sequences were classified as Archaea. Based on 97% similarity, a total of 3,731 OTUs ranging from 2,814 to 3,503 per sample were obtained for 16S rDNA (Fig. 1b).

### Diversity index analysis of microbial community

**Figure 2:**
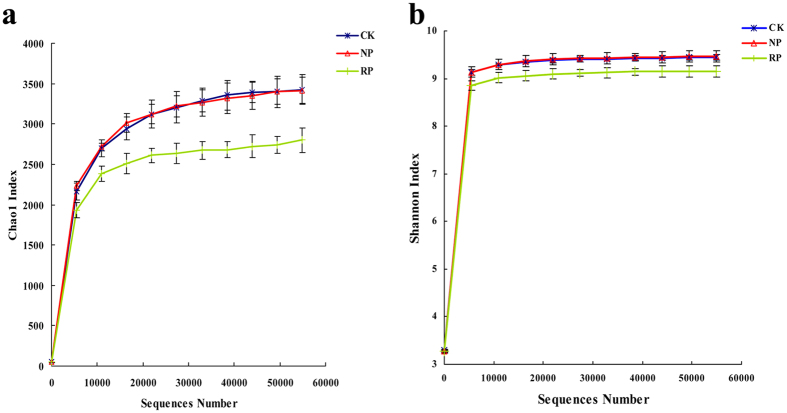
The Chao1 index curves (**b**), Shannon’s diversity index curves (**c**) based on 97% similarity. CK, RP, NP refer to control soil without planting any crop, newly planted soil, replanted soil, respectively. Data are representative of 3 independent experiments ± s.d. The figure is representative of 3 independent experiments.

Chao 1 and Lower Shannon index (Fig. 2a,b) after successive cropping of *P. hererophylla* indicated that the replanted *P. heterophylla* could reduce the abundance and diversity of the bacterial communities in rhizosphere soil.

### The analysis of microbial community structure and dominant genera

**Figure 3:**
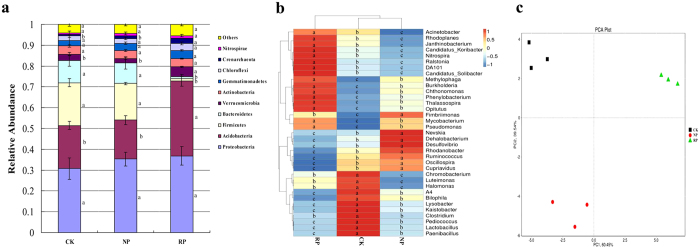
The analysis of the relative abundance of bacterial phyla (**a**), heatmap of the dominant genera distribution (**b**) and Principal component analysis (PCA) (**c**) of the three samples. CK, RP, NP refer to control soil without planting any crop, newly planted soil, replanted soil, respectively. (**a**) Data are representative of 3 independent experiments ± s.d. The figure is representative of 3 independent experiments. Statistical analysis was provided by student’s t-test, where ^a,b,c^*P* < 0.05. (**b**) The figure is representative of 3 independent experiments. Statistical analysis was provided by student’s t-test, where ^a,b,c^*P* < 0.05.

The successive cropping of *P. heterophylla* caused the significant change on the microbial composition and structure in rhizosphere (Fig. 3a and Supplementary Fig. S1). Compared with those in the newly planted soil, many bacteria species including *Verrucomicrobia*, *Acidobacteria*, *Crenarchaeota*, and *Chloroflexi* were sharply increased by 115%, 91.8%, 80.74%, and 46.7% in the replanted soil, respectively. Opposite was true in the case of *Firmicutes* and *Bacteroidetes* which showed significant decrease by 92.8%, and 89.6%, respectively. Moreover, at the phylum level, the replanted soil had significant difference in the microbial composition than the newly planted soil.

The analysis of the relative abundances of dominant genera (Fig. 3b) indicated that the successive cropping of *P. heterophylla* could cause the shifts in the structure of the microbial composition in the genus. Compared with those in the newly planted soil, the relative abundances of *Acinetobacter Chromobacterium*, *Thalassospira*, *Candidatus_Koribacter*, *Janthinobacterium*, *DA101*(one kind of bacterium genus name) etc. were increased sharply by 154.3%, 136.8%, 124.2%, 124.1% and 121.9% and 119.2%, respectively in the replanted soil and opposite was detected in *Dehalobacterium*, *Bilophila*, *Desulfovibrio*, *Oscillospira*, *Ruminococcus*, *Cupriavidus*, *Nevskia*, *Lactobacillus*, etc. indicating significant decrease by 100%, 100%, 100%, 99.5%, 98.6%, 90.7%, 42.4% and 27.1%, respectively in the replanted soil. Moreover, at genera level, the microbial composition in the control soil was much closer to that in the newly planted soil, while the microbial composition had a fairly large difference in the replanted soil and the newly planted soil.

The result of PCA (Fig. 3c) indicated that microbial community had a big difference in the control soil, newly planted soil and replanted soil. The successive cropping of *P. hererophylla* is a main factor influencing the microbial structure and diversity in rhizosphere. Overall, the continuous cropping of *P. hererophylla* could result in shifted microbial structure and decreased microbial diversity in rhizosphere soil.

### Ecological function analysis of microbial community

The results showed that most of harmful bacteria were significantly correlated with the increase of continuous cropping years positively, and inverse of this was found in the case of beneficial bacteria. The microbiome was mainly classified into five categories including carbon cycle, nitrogen cycle, sulfur cycle, probiotics and pathogens according to their ecological function in soil (Supplementary Table S1). In these identified bacteria, some bacteria were closely associated with soil texture, which function in the enhancement of the nutrient elements cycle in soil ecosystem, and some bacteria could improve the absorption of nutrients by plant, and hence promoted the growth of *P. heterophylla*. By contrast, most bacteria which were significantly positively correlated with the increase of continuous cropping years were *Acidobacteria*, denitrifying bacteria and pathogens, which are not conducive to plant growth in monoculture system (Supplementary Table S2).

Overall, the successive cropping of *P. heterophylla* could shift the diversity and structure of bacteria in rhizosphere soil of *P. heterophylla*, which showed that the diversity of bacteria in rhizosphere soil of *P. heterophylla* was decreased with the increase of planting years, while the structure of microbial community became more deteriorative. This finally resulted in the microecological imbalance in *P. heterophylla* rhizosphere, and caused serious consecutive monoculture problems as planting years extend.

### The DGGE analysis of fungi

**Figure 4:**
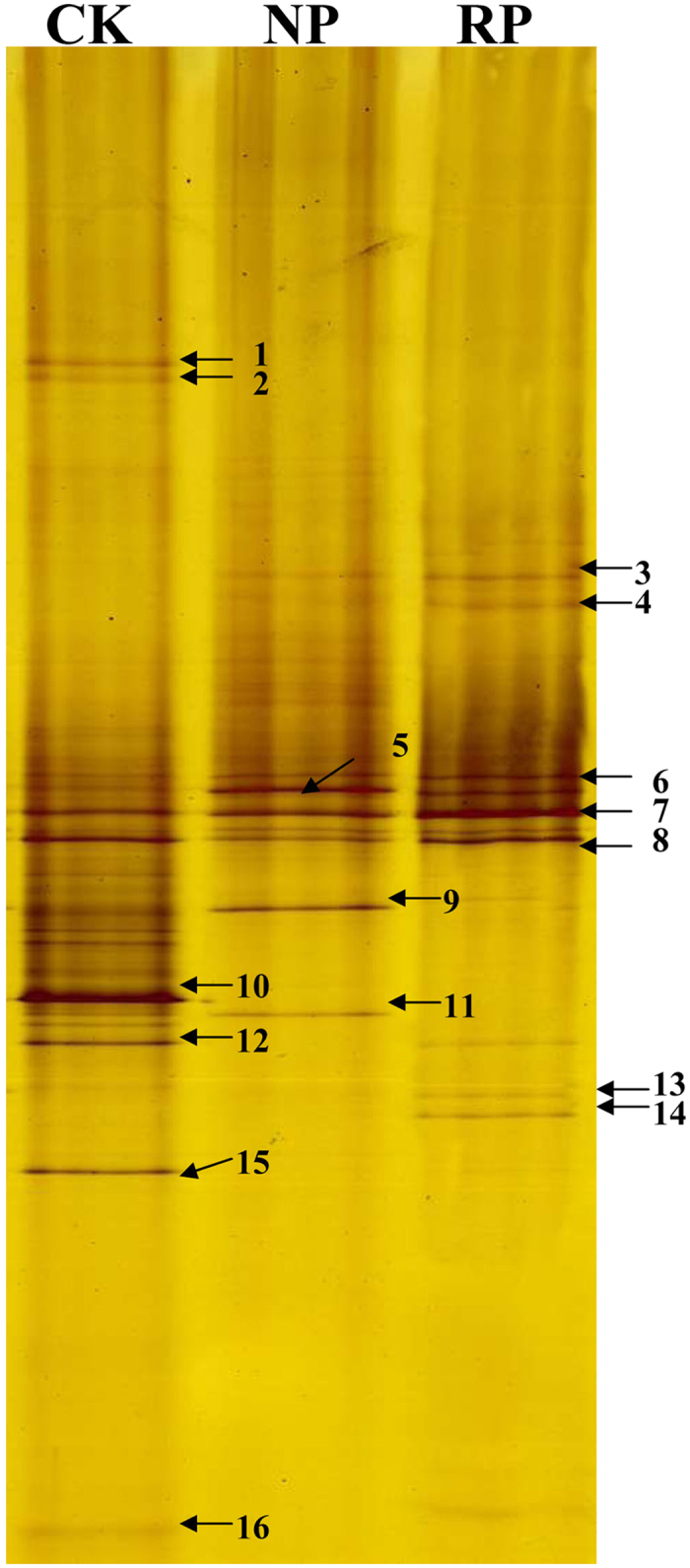
The fungi communities DGGE banding patterns of *P. hererophylla* rhizosphere soil of different cropping years. CK, RP, NP refer to control soil without planting any crop, newly planted soil, replanted soil, respectively. The figure is representative of 3 independent experiments.

Analysis of DGGE profiles showed that fractions from the control soil sample were the most, while fractions from replanted soil were the least (Fig. 4). These results suggested that rhizosphere fungi in the control soil were much more abundant than those in newly planted soil and replanted soil respectively.

The result in Supplementary Table S3 showed that 16 types of fungi had been identified successfully and was distributed to 13 species. Among them, seven kinds of fungi were known to function as pathogenic fungi, beneficial microbes, carbon cycling fungi and so on, while the other six kinds of fungi remained unknown in functions.

Overall, with continuously increasing cropping years, the structure of fungi had been changed in rhizosphere soil of *P. heterophylla*. The diversity of fungi in rhizosphere soil of *P. heterophylla* was decreased with the increase of planting years except that some specific pathogenic fungi were significantly increased in replanted soil. Our present study further confirmed that the abundance of some fungi was increased with the increase of planting years, as shown by their much more intense bands in DGGE profiles. These main pathogenic fungi, such as *F. oxysporum, Athelia rolfsii*, etc, and their pathogenicity were identified and validated, which could cause serious soil-borne disease during the period of monocropping *P. heterophylla*^21^. The findings suggested that structure dynamics of soil microbiota were mediated by the richness of replanted *P. heterophylla*, in turn led to microecological imbalance in *P. heterophylla* rhizosphere ecosystem and consequently caused serious consecutive monoculture problems in monocropping system.

### qPCR analysis of the specific pathogenic and beneficial microbes *in situ* rhizosphere soil of *P. heterophylla*

**Figure 5:**
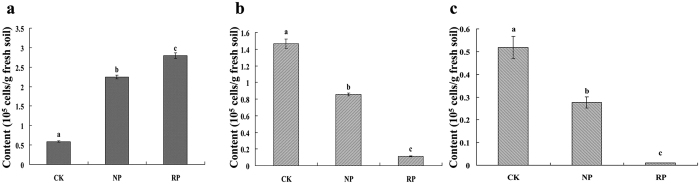
The content of *Fusaium oxysporum* f. sp. *hererophylla* (**a**), *Bacillus subtilis* (**b**) and *Bacillus amyloliquefaciens* (**c**) in *P. heterophylla* rhizosphere soils after different years of monoculture. CK, RP, NP refer to control soil without planting any crop, newly planted soil, replanted soil, respectively. Data are representative of 4 independent experiments ± s.d. The figure is representative of 4 independent experiments. Statistical analysis was provided by student’s t-test, where ^a,b,c^*P* < 0.05.

We first developed a standard curve of *B. subtilis* y = −2.6842x + 37.052 (R^2^ = 0.993) (Amplification efficiency E = 10^−1/slope^ = 2.358) and a standard curve of *B. amyloliquefaciens* y = −2.4131x + 37.742 (R^2^ = 0.986) (Amplification efficiency E = 10^−1/slope^ = 2.597) for our qPCR analysis. Results of qPCR analysis showed that the amount of *F. oxysporum* in the rhizosphere of *P. heterophylla* increased dramatically with the increase of cropping years (Fig. 5a). In contrast, we found that the amount of these two beneficial bacteria (*B. subtilis* and *B. amyloliquefaciens*) showed a sharp decrease under the monoculture system (Fig. 5b,c). The amount of *B. amyloliquefaciens* declined approximately 98% in soil after continuously being planted for two years. Analogously, the amount of *B. subtilis* decreased 93%. The changing pattern of the typical pathogen contrasted sharply with that of beneficial bacteria in the monocropping system, which further confirmed the result of DGGE analysis.

## Discussion

Consecutive monoculture problems, replant disease or soil sickness refer to a chemoecological phenomenon of plant dysplasia, serious diseases and significant decline in yield and quality caused by consecutively planting the same species in the same land for many years. Many crops suffer from the replant disease in modern cropping system^2^,^3^,^5^,^22^,^23^,^24^,^25^. Soil nutrient imbalance, autotoxins generation and/or change of soil microbial community structure are usually considered as fundamental factors of the soil sickness^21^. More recently, many researches suggested that microbes are the most important constituent of soil, and the changes in microbial community structure can influence the soil health which can affect the growth of plants directly^12^,^26^,^27^,^28^,^29^. In our previous study, we studied a typical pathogen *F. oxysporum* f.sp. *heterophylla*, which could increase sharply under the stimulation of root exudates from *P. heterophylla*, then resulted in the serious disease of *P. heterophylla* in a consecutive monoculture system^21^. However, *F. oxysporum* f.sp. *heterophylla* is only one of pathogens in root-associated microbial communities, the global analysis of the root-associated microbial communities can deliver an insight into the change of microbial communities in monoculture system. Accordingly, the systems biology approach including mategenome sequencing and DGGE were applied to study the changes of microbial community structure under a consecutive monoculture system of *P. heterophylla* for different years.

Results of bacterial mategenome sequencing showed that the microorganism community structure became worse, inferring that the population quantity of harmful microorganism increased while beneficial microorganism decreased with the increasing cropping years. The present study showed that the beneficial bacteria included the microorganisms participating in carbon cycle (such as *Dehalobacterium* and **Ruminococcus**), nitrogen cycle (such as**Desulfovibrio,* Cupriavidus, Rhodanobacter* and *Paenibacillus*) and sulfur cycle (*Desulfovibrio*) in soil. While these bacteria were found to be decreased with the increase in the cropping years and hence interdicted nutrient cycle in soil ecosystem. In addition, some microorganisms (such as **Cupriavidus,* Nevskia* and **Rhodanobacter**), functioning in degrading soil contaminant, were also decreased, and the changed soil conditions, as mentioned above were proved not to be conducive to the growth of the medicinal plants. These findings explain some of the reason of soil sickness popularly in modern intensive agriculture system. Our present result suggested that the soil metagenomic analysis based on 16S RNA gene sequences, was helpful to analyze the shifts of bacterial community in soil which accounts for 90% of microbial community and play important roles in the functioning of the soil ecosystem. However, it is not suitable for the analysis of fungi community due to the incomplete database, and in the years of study about *P. heterophylla*, we find that the fungi including beneficial fungi and pathogens in *P. heterophylla* rhizosphere are mainly a few species. So the DGGE method is enough for the study of fungi in consecutively monocropping system of *P. heterophylla.* Therefore, the method of DGGE was used for the present study. The results showed that the structure of fungi in rhizosphere soil of *P. heterophylla* had been modified under the monoculture system. In the replanted soil, the increased fungi were pathogens such as *F. oxysporum* and *A. rolfsii,* which could cause the wilt disease of *P. heterophylla*. Furthermore, in this study, qPCR analysis for the specific microbes can help to verify the veracity of mategenome sequencing and DGGE. Our results demonstrated that under the monoculture system, the changes in microbial community structure become adverse to the growth of *P. heterophylla*, showing that part of microbes participating in nutrient cycle of soil and the pathogens increased while the beneficial microbes decreased, which consequently caused serious consecutive monoculture problems in consecutively monocropping system. These results further confirmed our previous hypothesis that the consecutive monoculture problems or replant disease resulted from the changes of root-associated microbial communities^21^.

## Conclusions

In conclusion, our research reveals that the consecutive monoculture problem of *P. heterophylla* closely associates with the change of micro-ecology in rhizosphere ecosystem. The consecutive monoculture system of *P. heterophylla,* can cause the changes in the structure of microbial community, which ultimately results in the deterioration of microbial structure in rhizosphere soil with fewer beneficial microorganisms and more pathogenic microbes. This imbalanced rhizosphere microecological system, consequently leads to serious consecutive monoculture problems in continuous cropping system. This finding provides a clue to open a new avenue for modulating the root microbiome to enhance the crop production and sustainability.

## Materials and Methods

### The collection of plant and rhizosphere soil samples

The rhizosphere soil samples of *P. heterophylla* were collected from both newly planted and replanted fields in Zherong (119.89479E,27.23856N), which is the *P. heterophylla* Demonstration District of Agroecological Institute (Fujian Agriculture and Forestry University) at harvest time. The adjacent uncultivated field soil sample was used as a control. Five-point sampling method was used to collect the rhizosphere soil samples. Every treatment had three replicates. Soil samples were collected at the same time. *P. heterophylla* plants were carefully uprooted from the soil with a forked spade and slightly shaken to remove loosely attached soil. The rhizosphere soil tightly attaching to roots was brushed off and collected^30^. These soil samples were sieved using 2 mm sieve, then stored at −80 °C.

### Pathogenic fungus and beneficial bacteria isolation

*Fusarium oxysporum* f.sp. *heterophylla* and its two antagonistic bacteria (*Bacillus subtilis* and *Bacillus amyloliquefaciens*) were isolated from infected *P. heterophylla* plants in the *P. heterophylla* Demonstration District and provided by the Agroecological Institute, Fujian Agriculture and Forestry University.

### DNA extraction and purification

Total soil DNA from rhizospheric soil samples of *P. heterophylla* were extracted by using FastDNA^TM^ Spin Kit for Soil according to the manufacture’s instructions (MP Biomedicals, CA, USA). Different soil samples were used for DNA extraction. Every treatment had three replicates. DNA samples were then subjected to 1% agarose gel electrophoresis, recovered using Universal DNA Purification Kits to purify according to the manufacture’s instructions (TIANGEN BIOTECH (BEIJING) CO., LTD, China) and quantified by NanoDrop before being stored at −20 °C.

### The 16S rRNA amplicon metagenomic analysis of soil bacteria

Prepared DNA samples were sent to Novogene Bioinformatics Technology Co., Ltd (Beijing, China). According to the concentration, DNA was diluted to 1ng/μl using sterile water. Primer: 515F-806R (Table 1) targeting the bacteria and archaea 16S V4 region were selected for the microbial community structure analysis^31^, rRNA genes were amplified using the specific primer with the barcode. All PCR reactions were carried out in 30 μL reactions with 15 μL of Phusion^®^ High-Fidelity PCR Master Mix (New England Biolabs); 0.2 μM of forward and reverse primers, and about 10 ng templates DNA. PCR condition: fully denature 98 °C for 1 min, followed by 30 cycles of denaturation at 98 °C for 10 s, annealing at 50 °C for 30 s, and elongation at 72 °C for 60 s with a final extension at 72 °C for 5 min. Same volume of 1× loading buffer (contained SYB green) was mixed with PCR products and electrophoresis was operated on 2% agarose gel for detection. Samples with bright main strip between 400–450 bp were chosen for further experiments. PCR products were mixed in equidensity ratios. Then, PCR products were purified with GeneJET Gel Extraction Kit (Thermo Scientific). Sequencing libraries were generated using NEB Next^®^ Ultra™ DNA Library Prep Kit for Illumina (NEB, USA) following manufacturer’s recommendations and index codes were added. The library quality was assessed on the Qubit@ 2.0 Fluorometer (Thermo Scientific) and Agilent Bioanalyzer 2100 system. Every sample was with three replicates. At last, the library was sequenced on an Illumina MiSeq platform, and 250 bp/300 bp paired-end reads were generated. Every treatment had three replicates.

### Statistical and bioinformatics analysis

After removing the adaptor and primer sequences, the raw sequences were assembled for each sample according to the unique barcode using QIIME^31^. Paired-end reads from the original DNA fragments were merged using FLASH^32^. Paired-end reads were assigned to each sample according to the unique barcodes.

Sequences analysis was performed by UPARSE software package using the UPARSE-OTU and UPARSE-OTUref algorithms. In-house Perl scripts were used to analyze alpha (within samples) and beta (among samples) diversity. Sequences with ≥97% similarity were assigned to the same OTUs. We picked a representative sequences for each OTU and used the RDP classifier to annotate taxonomic information for each representative sequence. In order to compute Alpha Diversity, we rarified the OTU table and calculated three metrics: Chao1 estimates the species abundance; Observed Species estimates the amount of unique OTUs found in each sample, and Shannon index. Rarefaction curves were generated based on these three metrics.

Graphical representation of the relative abundance of bacterial diversity from phylum to species was visualized using Krona chart. Cluster analysis was preceded by principal component analysis (PCA). QIIME calculates both weighted and un-weighted unifrac distance.

### DGGE analysis of soil fungi

The previously purified DNA samples in this study were amplified by using PCR. The fungal DGGE primers were GCFung and NS1^33^ which amplified a suitable segment of SSU rRNA gene. PCR reactions were carried out in 50 μL reaction mixtures (containing 1 μL of each of the primers GCFung and NS1, DNA 1 μL, 2 × SYBR Green PCR Master Mix 25 μL, ddH_2_O 22 μL) for 40 cycles. With cycle conditions: fully denature at 94 °C for 3 min, followed by 40 cycles of 94 °C for 1min, 50 °C for 1min and 72 °C for 3 min, with a final extension at 72 °C for 10 min. The PCR products were detected using agarose gel and purified using TIAN pure Mini Plasmid Kit (TIANGEN BIOTECH (BEIJING) CO., LTD), then stored at 4 °C. Every sample had three replicates.

The DGGE technique was carried out using a Bio-Rad model 475 Gradient Delivery System (Hercules, Calif.) according to the manufacturer’s instructions Bio-Rad. The denaturing gradient was from 15% to 50%, and the concentration of acrylamide/bis (37.5:1) in the gel was 8%. The 100% denaturant solution contained 7 M urea, 40% (v/v) formamide. Following polymerization, PCR products 10 μL were mixed with an equal volume of 2× loading dye, and gels were run in 1× TAE at 60 °C for 20 h at 50 V. Gels were stained with silver staining, and photographed on the Bio-Rad Gel Doc 2000 Gel Documentation System. The scanned image of DGGE gel was analyzed with QuantityOne 4.0. Three replicates were taken for each sample.

The main bands were cut and transferred into sterile 1.5 mL EP tubes, then ddH_2_O (100 μL) was added. The samples were centrifuged at 12000 g, the supernatants were discarded, and the main bands were immersed in 100 μL sterile water at 4 °C overnight. Then centrifuged and the supernatants were transferred in new sterile 1.5 mL EP tubes. PCR reactions were carried out in 50 μL reaction mixtures (containing 1 μL of each of the primers Fung and NS1, the previous supernatants 3 μL, 2 × SYBR Green PCR Master Mix 25 μL, ddH_2_O 20 μL). Cycling conditions were as above. The PCR products were detected on agarose gel and sequenced by Shanghai biosune Co. Ltd (Shanghai, China). Then the results were compared with National Centre for Biotechnology Information (NCBI) gene bank to achieve the highest homology microbes. Every treatment had three replicates.

### Quantitative real-time PCR (qPCR) of *F. oxysporum* f.sp. *heterophylla* in rhizosphere soils under different year of monoculture systems

The previously purified DNA samples were amplified by using PCR, the primers YD1/YD2 for qPCR and product sizes were listed in Table 1. qPCR was operated as previously described^21^. Each treatment was applied with three replicates.

### Design of specific primers and PCR specificity analysis of *B. subtilis* and *B. amyloliquefaciens*

The sequencing information of the pathogenic strains was searched using BLAST and homologous comparison. Specific primers TJ-P1/TJ-P2 of *B. amyloliquefaciens* and TK-P1/TK-P2 of *B. subtilis* (Table 1) were designed using Premier Primer 5.0 software and synthesized from Shanghai Sangon Co. Ltd (Shanghai, China). The amplified target fragments were 219 bp and 137 bp in length, respectively.

### qPCR analysis of the specific pathogenic and beneficial microbes *in situ* rhizosphere soil of *P. heterophylla*.

The fragments were gel-purified, cloned into the PeasyTM-T4 Zero Cloning Kit (Beijing TransGen Biotech Co., Ltd.), and sequenced. The sequenced DNA was reamplified using TJ-P1/TJ-P2 and TK-P1/TK-P2 from the plasmid and purified using a TIAN pure Mini Plasmid Kit (TIANGEN BIOTECH (BEIJING) CO., LTD), respectively. The concentration of the target DNA samples were determined using a spectrophotometer (NanoDrop2000c, USA), and was then diluted to 5, 4, 3, 2, 1, 0.1, 0.01, and 0.001 ng/μL. qPCR was monitored on a Mastercycler ep realplex (Germany). Standard curve plotting and melting-curve analysis were performed following the qPCR amplification protocol^25^. The standard curve was created by plotting the target DNA concentration against the threshold cycle (Ct) value exported from the Mastercycler ep realplex. The primer sets TJ-P1/TJ-P2 and TK-P1/TK-P2 were evaluated using the established standard curve and melting curves were determined using qPCR amplification in four replicates with a serial dilution of the target DNA template. qPCR reactions were performed in 15 μL reaction mixtures (7.5 μL 2 × SYBR Green PCR Master Mix, 0.25 μL each primer, and 4 ng DNA made up to a final 15 μL with ddH_2_O). The qPCR parameters of *B. subtilis* and *B. amyloliquefaciens* were as follows: fully denature at 95 °C for 5 min, followed by 40 cycles of 95 °C for 10 s and 58 °C (TJ-P1/TJ-P2, TK-P1/TK-P2) for 20 s. After qPCR, melting curve analysis was performed to verify the specificity of the amplified product under the following conditions: 95 °C for 15 s, 60 °C for 15 s, followed by an increase to 95 °C over 10 min and a hold at 95 °C for 15 s. Every treatment had four replicates.

### Statistical Analysis

Analysis of variance was performed using DPS7.05. The differences in the means were determined by calculation of the least significant difference (LSD) at the 5% level.

## Additional Information

**How to cite this article**: Zhao, Y.P.Z. *et al.* Insight into structure dynamics of soil microbiota mediated by the richness of replanted *Pseudostellaria heterophylla*. *Sci. Rep.* **6**, 26175; doi: 10.1038/srep26175 (2016).

## Supplementary Material

Supplementary Information

## Figures and Tables

**Table 1:** DNA sequences of the primer pairs used in this study.

Primer name	Sequence (5′ → 3′)	Fragment size (bp)
NS1	GTAGTCATATGCTTGTCTC	350 bp
Fung	ATTCCCCGTTACCCGTTG	
GC Fung	CGCCCGCCGCGCCCCGCGCCCGGCCCGCCGCCCCCGCCCC ATTCCCCGTTACCCGTTG	
515F	GTGCCAGCMGCCGCGGTAA	250 bp
806R	GGACTACHVGGGTWTCTAAT	
YD1	GCAGCGAGACCGCCACTAGATTT	106 bp
YD2	TGCCTGTTCGAGCGTCATTTCA	
TJ-P1	GCAGCGAGACCGCCACTAGATTT	219 bp
TJ-P2	ACTTTACTGCGAGCTTGTCCGT	
TK-P1	GCGGAATCATCCGTATTGGGGCAGA	137 bp
TK-P2	AACCTCGCGGGCTTTCTCGCCAA	
