# Supplementary material for: Insight into structure dynamics of soil microbiota mediated by the richness of replanted Pseudostellaria heterophylla
Source: Sci Rep. 2016 May 18;6:26175. doi: 10.1038/srep26175 (PMC4870612; doi:10.1038/srep26175)
Supplement: Supplementary Information [file srep26175-s1.doc]

**Title**

Insight into structure dynamics of soil microbiota mediated by the richness of replanted *Pseudostellaria heterophylla*.

**Authors and e-mail address**

Yong-Po Zhao ^1,2^, Sheng Lin ^1,2^, Leixia Chu^1, 3^, JiangTao Gao^1,2^, Saadia Azeem^1,3^, Wenxiong Lin ^1,2*^ ,

*Corresponding author

**Affiliations**

1. College of Life Sciences, Fujian Agricultural and Forestry University, Fuzhou 35002, China;

2. Key Laboratory of Crop Ecology and Molecular Physiology, Fujian Agriculture and Forestry University, Fuzhou 35002, China

3. Fujian Provincial Key Laboratory of Agroecological Processing and Safety Monitoring, Fujian Agriculture and Forestry University, Fuzhou 35002, China

**Full address for correspondence**

*Corresponding author: Wenxiong Lin, Agroecological Institute, Fujian Agriculture and Forestry University, Fuzhou 35002, Fujian, People's Republic of China.

**Phone** +86-591-83737535  **Fax** +86-591-83769440

**E-mail address** 30403006@fafu.edu.cn

**
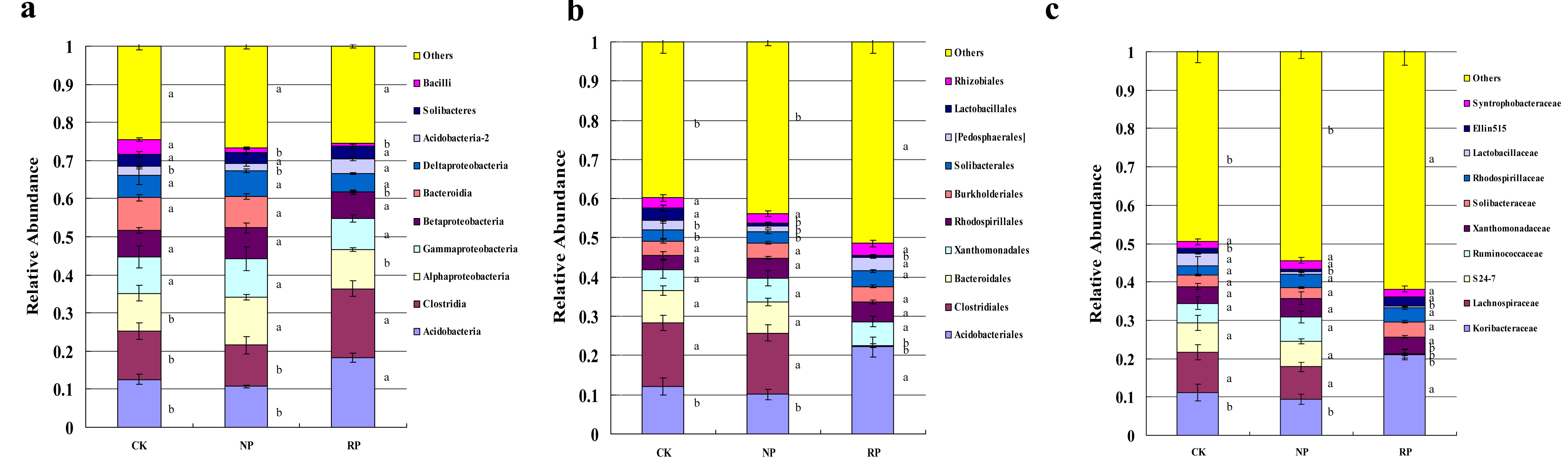
**

Supplementary Figure S1. **Comparison of the bacterial communities at the** **class (a), order (b) and family (c) level.** CK, NP and RP refer to the control soil without planting any crop, newly planted soil and replanted soil, respectively. Sequences that could not be classified into any known group are labeled ‘‘other’’. Data are representative of 3 independent experiments±s.d.. The figure is representative of 3 independent experiments. Statistical analysis was provided by student’s t-test, where ^a,b,c^ *P*＜0.05.

**Supplementary Table** **S1. The bacteria which had a significant negative correlation with the increase of continuous cropping years**

| **Representative population（negative correlation）** | **Main Ecological Function** | **Rate of decrease (%)** |
| --- | --- | --- |
| *Dehalobacterium* | carbon cycle[^1^](#_ENREF_1), dehalogenation | 100.00% |
| *Bilophila* | unknown | 100.00% |
| *Desulfovibrio* | sulfur cycle[^4^](#_ENREF_4) , nitrogen cycle[^5-7^](#_ENREF_5) | 100.00% |
| *Oscillospira* | carbon cycle [^8^](#_ENREF_8), degrade dimethyl sulfide[^9^](#_ENREF_9) | 99.48% |
| *Ruminococcus* | carbon cycle[^10-13^](#_ENREF_10) | 98.62% |
| *Cupriavidus* | carbon cycle[^14^](#_ENREF_14), nitrogen cycle[^15^](#_ENREF_15), degrade soil contaminant[^16^](#_ENREF_16) | 90.65% |
| *Nevskia* | degrade soil contaminant[^17^](#_ENREF_17) | 42.40% |
| *Lactobacillus* | beneficial bacteria[^18^](#_ENREF_18), degrading organic matter[^19^](#_ENREF_19) | 27.05% |
| *Pediococcus* | carbon cycle [^20^](#_ENREF_20), beneficial bacteria[^21^](#_ENREF_21) | 22.03% |
| *A4* | unknown | 21.85% |
| *Rhodanobacter* | degrade soil contaminant  nitrogen cycle | 16.67% |
| *Paenibacillus* | nitrogen cycle[^26^](#_ENREF_26)  beneficial bacteria[^26-29^](#_ENREF_26) | 9.09% |
| *Fimbriimonas* | carbon cycle[^30^](#_ENREF_30) | 8.70% |
| *Kaistobacter* | unknown | 3.38% |
| *Lysobacter* | beneficial bacteria[^31-33^](#_ENREF_31)  degrade soil contaminant[^34^](#_ENREF_34) | 2.72% |

**Rate of decrease (%) = (**the relative abundances in control soil without planting any crop - the relative abundances in replanted soil**)/** the relative abundances in control soil without planting any crop×100%

Supplementary Table S2. **The bacteria which had a significant positive correlation with the increase of continuous cropping years**

| **Representative population（positive correlation）** | **Main Ecological Function** | **Rate of increase (%)** |
| --- | --- | --- |
| *Mycobacterium* | degrade polycyclic aromatic hydrocarbon[^35^](#_ENREF_35) | 1.47% |
| *Pseudomonas* | plant growth promoting  rhizobacteria [^36-38^](#_ENREF_36) or pathogen[^39^](#_ENREF_39) | 7.27% |
| *Rhodoplanes* | nitrogen cycle (denitrification)[^40^](#_ENREF_40), Phototrophic bacteria | 15.75% |
| *Phenylobacterium* | degrade soil contaminant | 15.95% |
| *Opitutus* | unknown | 17.31% |
| *Luteimonas* | nitrogen cycle (denitrification) | 18.75% |
| *Ralstonia* | pathogen[^47^](#_ENREF_47) | 21.18% |
| *Chthonomonas* | carbon cycle [^30^](#_ENREF_30) | 31.96% |
| *Candidatus-Solibacter* | acidophilic bacteria | 38.84% |
| *Burkholderia* | beneficial bacteria , pathogen[^52^](#_ENREF_52) | 42.28% |
| *Methylophaga* | unknown | 50.57% |
| *Halomonas* | nitrogen cycle (denitrification), degrade aromatic compounds[^54^](#_ENREF_54) | 96.40% |
| *Nitrospira* | nitrogen cycle | 119.20% |
| *DA101* | unknown | 121.20% |
| *Janthinobacterium* | pathogen | 121.90% |
| *Candidatus**-Koribacter* | acidophilic bacteria | 124.10% |
| *Thalassospira* | pathogen[^60^](#_ENREF_60), nitrogen cycle (denitrification)[^61^](#_ENREF_61) | 124.20% |
| *Chromobacterium* | pathogen | 136.80% |
| *Acinetobacter* | acidophilic bacteria[^64^](#_ENREF_64) , plant growth promoting rhizobacteria [^65^](#_ENREF_65) | 154.30% |

**Rate of increase (%) = (**the relative abundances in replanted soil - the relative abundances in control soil without planting any crop**)/** the relative abundances in control soil without planting any crop×100%

Supplementary Table S3. **The identification and functional analysis of fungi in rhizosphere soil of different-years of planting *P. heterophylla***

| numerical order | Representative population | Main Ecological Function |
| --- | --- | --- |
| 1 | *Achaeta camerani* | Unknown |
| 2 | *[Achaeta unibulba](http://blast.ncbi.nlm.nih.gov/Blast.cgi#alnHdr_316990119)* | Unknown |
| 3 | *Gibberella intermedia* | pathogenic fungi[^66^](#_ENREF_66) |
| 4 | *Uncultured Fusarium sp* | pathogenic fungi or saprophytic fungi[^67^](#_ENREF_67) |
| 5 | *[Trichoderma reesei strain](http://blast.ncbi.nlm.nih.gov/Blast.cgi#alnHdr_408683830),* | saprophytic fungi[^68^](#_ENREF_68), carbon cycle[^69^](#_ENREF_69) |
| 6 | *[Cryptococcus humicolus](http://blast.ncbi.nlm.nih.gov/Blast.cgi#alnHdr_8698566)* | carbon cycle |
| 7 | *[Fusarium oxysporum](http://blast.ncbi.nlm.nih.gov/Blast.cgi#alnHdr_590123891)* | pathogenic fungi[^72^](#_ENREF_72) |
| 8 | *Athelia rolfsii* | pathogenic fungi[^73^](#_ENREF_73) |
| 9 | *[Uncultured fungus](http://blast.ncbi.nlm.nih.gov/Blast.cgi#alnHdr_399573266)* | Unknown |
| 10 | *Hemienchytraeus sp.* | Unknown |
| 11 | *Uncultured fungus* | Unknown |
| 12 | *Rhyacodrilus coccineus* | Unknown |
| 13 | *Enchytraeidae sp.* | Unknown |
| 14 | *[Cladosporium sp.](http://blast.ncbi.nlm.nih.gov/Blast.cgi#alnHdr_403404998)* | pathogenic fungi[^74^](#_ENREF_74) |
| 15 | *Uncultured fungus* | Unknown |
| 16 | *Uncultured fungus* | Unknown |

**References**
